# Advances in therapeutic and managemental approaches of bovine mastitis: a comprehensive review

**DOI:** 10.1080/01652176.2021.1882713

**Published:** 2021-02-17

**Authors:** Khan Sharun, Kuldeep Dhama, Ruchi Tiwari, Mudasir Bashir Gugjoo, Mohd. Iqbal Yatoo, Shailesh Kumar Patel, Mamta Pathak, Kumaragurubaran Karthik, Sandip Kumar Khurana, Rahul Singh, Bhavani Puvvala, Amarpal, Rajendra Singh, Karam Pal Singh, Wanpen Chaicumpa

**Affiliations:** a Division of Surgery, ICAR-Indian Veterinary Research Institute, Izatnagar, Bareilly, Uttar Pradesh, India; b Division of Pathology, ICAR-Indian Veterinary Research Institute, Izatnagar, Bareilly, Uttar Pradesh, India; c Department of Veterinary Microbiology and Immunology, College of Veterinary Sciences, Deen Dayal Upadhayay Pashu Chikitsa Vigyan Vishwavidyalay Evum Go-Anusandhan Sansthan (DUVASU), Mathura, India; d Division of Veterinary Clinical Complex, Faculty of Veterinary Sciences & Animal Husbandry, Sher-E-Kashmir University of Agricultural Sciences and Technology of Kashmir, Shalimar, Srinagar, Jammu and Kashmir, India; e Sher-E-Kashmir University of Agricultural Sciences and Technology of Kashmir, Shalimar, Srinagar, Jammu and Kashmir, India; f Central University Laboratory, Tamil Nadu Veterinary and Animal Sciences University, Chennai, Tamil Nadu, India; g ICAR-Central Institute for Research on Buffaloes, Hisar, Haryana, India; h Rajiv Gandhi Institute of Veterinary Education and Research, Kurumbapet, Puducherry, India; i Center of Research Excellence on Therapeutic Proteins and Antibody Engineering, Department of Parasitology, Faculty of Medicine Siriraj Hospital, Mahidol University, Bangkok, Thailand

**Keywords:** bovine, cow, welfare, mastitis, etiology, management, treatment, therapeutics, review

## Abstract

Mastitis (intramammary inflammation) caused by infectious pathogens is still considered a devastating condition of dairy animals affecting animal welfare as well as economically incurring huge losses to the dairy industry by means of decreased production performance and increased culling rates. Bovine mastitis is the inflammation of the mammary glands/udder of bovines, caused by bacterial pathogens, in most cases. Routine diagnosis is based on clinical and subclinical forms of the disease. This underlines the significance of early and rapid identification/detection of etiological agents at the farm level, for which several diagnostic techniques have been developed. Therapeutic regimens such as antibiotics, immunotherapy, bacteriocins, bacteriophages, antimicrobial peptides, probiotics, stem cell therapy, native secretory factors, nutritional, dry cow and lactation therapy, genetic selection, herbs, and nanoparticle technology-based therapy have been evaluated for their efficacy in the treatment of mastitis. Even though several strategies have been developed over the years for the purpose of managing both clinical and subclinical forms of mastitis, all of them lacked the efficacy to eliminate the associated etiological agent when used as a monotherapy. Further, research has to be directed towards the development of new therapeutic agents/techniques that can both replace conventional techniques and also solve the problem of emerging antibiotic resistance. The objective of the present review is to describe the etiological agents, pathogenesis, and diagnosis in brief along with an extensive discussion on the advances in the treatment and management of mastitis, which would help safeguard the health of dairy animals.

## 1. Introduction

Mastitis is a disease of high impact on animal welfare as well as on economy among bovine diseases. It adversely affects the lucratively benefits of animal producers/farmers and leads to a large production loss in the dairy sector worldwide (Bardhan 2013; Sinha et al. 2014; Izquierdo et al. 2017; Aghamohammadi et al. 2018; Das et al. 2018). Bovine mastitis is the inflammation of the mammary glands/udder (intramammary inflammation, IMI) in cows. The disease is caused mainly by bacterial infections and is classified into two types based on epidemiology, namely contagious and environmental mastitis (Garcia 2004). The former is caused by contagious bacteria including *Staphylococcus aureus* (*S. aureus*), *Streptococcus agalactiae* (*S. agalactiae*) and *Mycoplasma* spp. that are spread from infected cow to a healthy cow usually at the time of milking through the hands, towels and/or the milking machine acting as the bacterial reservoirs. Conversely, environmental mastitis is caused by bacteria that spread primarily outside of the milking parlor, *i.e.,* the causative bacteria come from the cow’s environment such as bedding material, soil, manure, feces, and stagnant water (Garcia 2004). Bovine mastitis causes an increase in the cost of rearing as far as the production of milk is concerned. Moreover, it has also a negative effect on the composition of the milk as well as its value (Halasa et al. 2007; Kalinska et al. 2017). Environmental mastitis is highly influenced by management practices (Garcia 2004); and thus, requires better technical and biological tools along with suitable encouragement and incentives. Farmers and field veterinarians need to work in accordance with official guidelines for use of mandated antimicrobials (Klaas and Zadoks 2018). During the last century, significant advances to control mastitis have been made; but due to the changing population dynamics, herd structure and more rigorous processor standards that make mastitis a complicated disease and remains a foremost problem of the dairy industry. Thus, further extensive research in the area is demanded (Ruegg 2017a).

In cattle and buffalo, mastitis is an important economic problem worldwide including India (Das et al. 2018), Canada (Aghamohammadi et al. 2018), Germany (Hamann 2001), United Kingdom (Bennett et al. 1999), the Netherlands (Hogeveen et al. 2011) and United States of America (Hadrich et al. 2018). Bovine mastitis is associated with a daily loss that varied from 1.0 to 2.5 kg of milk during the first two weeks following the onset, and a total loss of 110 to 552 kg during the entire lactation depending upon the parity and time of occurrence. Mastitis also has a long-lasting effect on the milk yield, since cows will not regain its peak milk yield in their remaining part of the lactation (Rajala-Schultz et al. 1999). Despite various advanced management practices in cattle and buffalo rearing in the dairy sector, mastitis is still a daunting disease and among the major economic issues of farmers and dairy owners. India is ranked on the top among milk producing countries (cattle and buffalo milk combined). The economic loss due to mastitis in India is about Rs. 575 million per annum and it reduces milk by 21% (Bardhan 2013). In addition, the consumption of mastitis-affected milk maybe harmful to humans as antimicrobial resistant pathogens may be transmitted by contaminated unpasteurized milk; hence is also a major public health concern/hazard (Oliver and Murinda 2012). In addition, health hazards related to increased microbial resistance and residues of antibiotics in milk resulted in increased consumer demand for organic products as they believe foods produced through conventional farming systems are healthier and safer for consumption (Ruegg 2009). Due to zoonotic threats, mastitis milk cannot be consumed and also cannot be sold; thus, contributing to major economic losses. Infected udder reduces the price of animals in the market and exerts an economic burden on the owner from the costs of treatment (González and Wilson 2003; Seegers et al. 2003).

Though the association between mastitis and pathogenic microorganisms was established in 1887, the major pathogens were identified only during the 1940s. The finding on multi-factorial etiology of bovine mastitis in 1960s paved way for further research into mastitis (Singh and Singh 1994; Ndlela et al. 2016) including identification of the common etiologic agents which were found to be both Gram-positive and Gram-negative bacteria such as *S. agalactiae*, *S. aureus*, *Escherichia coli* (*E. coli*) and *Klebsiella pneumoniae* (*K. pneumoniae*)*;* molecular epidemiology of the causative pathogens; comparative methods for typing the pathogens at the subspecies level; virulence gene arrays; whole genome sequencing and investigations of *in vitro* antibiotic susceptibility pattern. As time progressed, antibiotic (penicillin) therapy was made available by 1945, but it was not effective against all pathogens causing mastitis. There is a need for management practices to target the pre-calving period in heifers in order to reduce the probability of mastitis in later stages (Naqvi et al. 2018). Generally, subclinical mastitis and IMIs in heifers during calving is predominantly caused by major pathogens, *viz*., coagulase-negative staphylococci leading to heifer mastitis. In early lactation, IMIs are influenced by many factors including nature of the disease, virulence of the pathogen, onset time in relation to calving, persistence of the infection/cure, host immunity, stage of pregnancy and managemental practices including risk associated with season and herd location. A short-term prepartum antibiotic treatment is an effective measure to control heifer mastitis, but it is rarely recommended because of the long-lasting adverse effects on udder health and milk production, thereby lowering the profit of the farmers (De Vliegher et al. 2012).

Diagnosis of mastitis is the major requirement of the dairy industry for clean milk production, not only for economical reasons and public health concerns, but also regarding animal welfare. Diagnosis needs to be early, rapid and accurate for prevention of mastitis or early detection of mastitis for management or therapeutic purposes. This envisages application of conventional as well as advanced diagnostic tests. The conventional methods are relatively cheap, easy, rapidly available, and field applicable, but usually non-specific. The advanced tests are costly, requiring technical skill and sophisticated infrastructure and facilities, but usually accurate and specific for different forms of mastitis (Swarup et al. 1989; Singh et al. 2013; Hussein et al. 2018; Chakraborty et al. 2019).

Blanket dry cow therapy, strategic culling and well-defined biosecurity protocols are effective measures to control and prevent the reintroduction of other virulent strains of *S. agalactiae* and *S. aureus* (Kefee 2012). In addition, combination of antibiotic treatment and culling of unresponsive cows showed decline in transmission rate and reduction in IMIs (Halasa 2012). Several modes of conventional and advanced therapeutic measures are available for the management of mastitis, which include antibiotics, vaccination, nanoparticle-based therapy, herbal therapy, and bacteriocins (Gomes and Henriques 2016). Various agents contribute to the reduction in udder infections particularly mastitis in cows and also aid in improvement of the milk quality (Skowron et al. 2019). Among these, antibiotic therapy and vaccination are the commonly used methods for the treatment of mastitis. Extensive and uncontrolled use of antibiotics for the treatment along with the induction and persistence of biofilm-associated antibiotic resistance in mastitis has led to the decreased response to antibiotic therapy (Park et al. 2012; Babra et al. 2013). Although vaccination is ineffective against bovine mastitis because a variety of microorganisms are involved in its development; nevertheless, *S. aureus*, *Streptococcus uberis* (*S. uberis*), and *E. coli* were thought to be the major targets for vaccine development (Wilson et al. 2009; Bradley et al. 2015; Collado et al. 2016; Ashraf and Imran 2020). Even though several commercial vaccines are available, most of them failed to demonstrate sufficient protection and at the same time are costly (Côté-Gravel and Malouin 2019). Due to these shortcomings of the antibiotics and vaccines, several other modes of therapy have emerged that can fill the lacunae. Nanoparticle technology and bacteriocins (antimicrobial peptides) are few advanced therapeutic agents or techniques that are promising for the prevention of mastitis (Castelani et al. 2019; Godoy-Santos et al. 2019; Pinheiro Machado et al. 2019).

The present review describes various aspects of mastitis/intramammary infections (IMIs) with a special focus on its etiology, brief scenario, diagnosis, management and advances in treatment and developing novel therapeutics for countering this important disease affecting bovine population/dairy herds.

## 2. Mastitis causing pathogens

Majority of the pathogens that cause clinical bovine mastitis are from environmental or ubiquitous origin. In contrast, contagious agents are mainly related to subclinical infections (Abebe et al. 2016; Klaas and Zadoks 2018). Mastitis is a multi-etiological disease and some bacteria are mainly responsible for clinical, subclinical, contagious, and environmental mastitis. The most common bacteria involved are *S. aureus, S. agalactiae, S. pyogenes, Trueperella pyogenes* (*T. pyogenes*)*, E. coli, K. pneumoniae, K. oxytoca, Enterobacter aerogenes, Pasteurella* spp. (Levison et al. 2016; Abdalhamed et al. 2018; Shinozuka et al. 2018; Zhang et al. 2018). Among these contagious pathogens, *S. aureus, S. dysgalactiae* and *S. agalactiae*. *S. aureus* are the predominant organisms while major environmental pathogens are members of Enterobacteriaceae particularly *E. coli* and also *S. uberis* (Petersson-Wolfe et al. 2010). *S. agalactiae* is the most common Gram-positive bacterium from clinical mastitis, followed by *S. aureus* while *Klebsiella* spp. and *E. coli* were the most isolated Gram-negative bacteria from clinical mastitis (Cortinhas et al. 2016). *S. agalactiae* and *S. aureus* primarily spread through contact, so herd biosecurity may be considered as an important preventive measure for reduction and elimination of the reservoirs (Kefee 2012).

Both clinical and subclinical forms of mastitis can be caused by most of the bacterial pathogens. However, *T. pyogenes* is responsible for causing solely clinical form of mastitis (Malinowski et al. 2006). In primiparous cows, the highest loss of milk is due to *S. aureus*, *Klebsiella* spp. as well as *E. coli*. In older cows, significant losses are due to infections by *Streptococcus* spp., *T. pyogenes, S. aureus, Klebsiella* spp., and *E. coli* (Gröhn et al. 2004). In general, *S. aureus, S. agalactiae,* and *S. uberis* are common pathogens causing mastitis, whereas *Mycoplasma bovis* and *Corynebacterium bovis* are less frequently involved (Wernicki et al. 2014; Vakkamäki et al. 2017). Coagulase-negative staphylococci and their role in causing mastitis should also be seriously taken into consideration (Krukowski et al. 2001). Wilson et al. (1997) have reported that *S. agalactiae* along with various pathogens including *Prototheca* sp., *Streptococcus* spp. and *T. pyogenes* are associated with majority of the cases of mastitis. Mastitis occurs in its heaviest form when associated with infections due to coliforms, CAMP-negative *Streptococcus* spp., *T. pyogenes*, *S. agalactiae*, fungi (yeast-like) and *Prototheca* sp. (Wilson et al. 1997; Malinowski et al. 2006). *Corynebacterium* spp. (40%) and *S. aureus* (32%) were the most common isolates found by Steele and McDougall (2014) in cases of sub-clinical mastitis in New Zealand. *Prototheca* spp. are pathogenic algae and opportunistic pathogens that cause mastitis in dairy herds and possess zoonotic potential (Alves et al. 2017; Dos Anjos et al. 2019).

*S. aureus* has been the most common pathogen identified in mastitis (McParland et al. 2019). Methicillin resistant-*S. aureus* (MRSA) CC22-MRSA-IV has been reported as intramammary pathogen by Magro et al. (2018). On genotyping by DNA microarrays, MRSA was noted as epidemic UK-EMRSA-15 grouping in CC22. These isolated strains had genes for β-lactam and macrolide resistance. Isolates were obtained from milkers and dairy cows, hence suggesting reverse zoonosis. Routine sampling and evaluation of milk has identified the presence of mastitis causing pathogens in 13% of the total samples collected from dairy herds. Among the isolated pathogens, *S. aureus*, *Streptococcus* spp., *T. pyogenes* and *C. bovis* were found to be the most common pathogens (Cvetnić et al. 2016).

## 3. Types of mastitis and clinical relevance

Mastitis can be epidemiologically categorized into contagious and environmental mastitis and are caused by a wide spectrum of pathogens. In addition to that, mastitis can also be classified as either clinical or subclinical form (Garcia 2004; Abebe et al. 2016). Any increase in the level of humidity and pollution in the environment of the barn also increases the load of bacterial pathogens in the environment. One of the studies has shown 74.7% prevalence of mastitis at herd-level while 62.6% prevalence at cow level. In relation to the subclinical and clinical mastitis, the former type appears to be responsible for the majority of cases (59.2%) than the latter (3.4%) (Abebe et al. 2016). Clinical mastitis can be identified easily based upon visible symptoms in terms of udder inflammation showing redness in affected part or complete udder, warmth, swelling, pain upon touch, milk clots, discolouration and change in consistency of milk. The general symptoms are pyrexia (> 39.5 °C) and loss of appetite.

The major causes of clinical mastitis are caused by environmental pathogens that include coliforms. Among 20,000 clinical mastitis cases in the Netherlands, 40% were caused by *S. uberis* and *S. dysgalactiae*, 30% by *S. aureus* and 30% by *E. coli* (Steeneveld et al. 2011). The udder of the cow may show decreased susceptibility as well as resistance to inflammatory condition during certain conditions. Such conditions include administration of antibiotic through the udder for prolonged period of time, higher incidence of occurrence of mycosis of udder resulting from deficiency of mineral-vitamin and of antioxidant, dietary imbalance, and poor conditions of the environment as well as changes in weather (Wawron et al. 2010).

Kumar et al. (2010) studied incidence and economical aspects of clinical mastitis. As compared to clinical form, no clinically visible symptoms appear in subclinical mastitis, although change in milk composition can be an indicator. Hence, it is recognized and confirmed by laboratory examination of milk or by animal-side tests such as California mastitis test (CMT) followed by laboratory isolation of the causal agent.

The somatic cell count (SCC) in the milk should be less than 200,000 per mL in the healthy cow. Somatic cells are mostly white blood cells (WBCs), *viz*., infiltrated neutrophils as well as macrophages into the tissue of the mammary gland due to inflammation (Akers and Nickerson 2011). *S. agalactiae* mainly localizes in the udder and causes persistent infections with higher SCC (Kefee 2012).

Mastitis is a result of host immune response to infectious agents affecting the udder (Gurjar et al. 2012). Usually there is a balance of microflora in a healthy udder. The intramammary microbiota is composed of complex community of diverse bacteria (Rainard 2017; Andrews et al. 2019). The commensal mammary microbiota present in the healthy udder plays a major role in the immune homeostasis (Derakhshani et al. 2018). Therefore, a disruption in the diversity of udder microbiota (dysbiosis) can have an impact on mastitis. The normal microbiome of udder is an important factor to be kept in consideration when making diagnosis of mastitis as healthy quarters also contain some bacteria. Various bacterial genera such as *Ruminococcus, Oscillospira, Roseburia, Dorea, Prevotella, Bacteroides, Paludibacter,* and *Bifidobacterium* are usually present in the udder. Any assault or congenital anomaly of the udder or teat, such as teat fistula, teat spider, leaky teat, and udder wounds that exposes the udder to the external microbes or retention of milk tends to cause mastitis (Rambabu et al. 2011).

In a study, the mastitis-affected tissue revealed marked inflammation with notable reductions in the alveolar epithelium and lumen whereas increment in stromal connective tissue along with leukocytosis was reported histopathologically (Nickerson et al. 1995). These types of affections either expose the udder to external pathogens or weaken the internal resistance. In clinical form of mastitis, mostly *Staphylococcus* spp. or *E. coli* are prevalent and the normal microbiota is disturbed. Researchers have proposed that either due to alteration in the normal microbiome because of pathogens or prolonged use of antibiotic therapy mastitis begins and establishes (Falentin et al. 2016). In a detailed molecular epidemiological study, majority of the dairy cattle in the USA were found to carry more than 10 coagulase-negative *Staphylococcus* (CNS) species and the bacteria were isolated at different stages of lactation (Jenkins et al. 2019).

Mastitis is a complex detrimental outcome of various factors working together at the host level. These involve pathogens, their growth pattern in the udder parenchyma, signalling pathways for establishing the clinical manifestations, and various molecular mechanisms mediated by pathogen-associated molecular patterns (PAMPs). This is made possible *via* different pattern recognition receptors (PRRs) of the host, such as Toll-like receptors (TLRs), NOD-like receptors (NLRs) and RIG-like receptors (RIGs), in instigating the udder inflammation due to microbial infections, along with an array of environmental factors. Hence, demands collaborative approach for diagnosis as well as control of this important disease (Bhattarai et al. 2018).

## 4. Economic significance

Both clinical and subclinical mastitis causes loss of milk as it has to be discarded apart from the cost of treatment and other expenses (Halasa et al. 2007; Kumar et al. 2010). Subclinical mastitis causes three times more production losses than clinical mastitis; thus, is responsible for significant losses of 60-70% of the total economic losses associated with all mastitis due to infections (De Vliegher et al. 2012; Sinha et al. 2014). Sinha et al. (2014) performed study on dairy animals to assess the incidence and economics of subclinical form of bovine mastitis in Central Region of India. The losses due to mastitis were approximately 49% due to loss of value of milk and 37% owing to veterinary expenses. The cost of treating an animal includes cost of medicine (31%) and services (5.5%). Losses were comparatively higher in cross-bred cattle due to their high production potential that was affected during mastitis period.

## 5. Advances in diagnosis of mastitis

Conventional diagnostic tests for mastitis are usually qualitative with lesser specificity and sensitivity, whereas advanced tests are quantitative, highly specific and sensitive (Godden et al. 2017; Hussein et al. 2018; Chakraborty et al. 2019). Advanced molecular techniques based on phenotyping as well as genotyping methods offer rapid and specific identification methods for diagnosis of mastitis causing pathogens up-to species and subspecies level (Gurjar et al. 2012). It is important to determine the species of bacteria for proper selection of antibiotic for therapeutic purpose and for selecting proper processing method to produce dairy products specifically. Systems of identification (automated), *viz*., VITEK identification cards are available for this purpose providing results of identification of bacteria with stability (Harjanti et al. 2017). Kandeel et al. (2018) opined that all dairy cattle which are admitted to any veterinary hospital should be managed in a way that they are having intramammary infection even when there is no overt clinical mastitis. Various advances in diagnostics applicable for timely and accurate detection of mastitis have been reviewed recently by Chakraborty et al. (2019) and included phenotyping or general type of tests and genotyping or specific type of tests. The former comprises of tests based on physico-biochemical, non-specific cultural and proteomics, while the latter includes a specific culture, polymerase chain reaction (PCR) and its various versions (*e.g.,* qRT-PCR) (Behera et al. 2018), loop-mediated isothermal amplification (LAMP), lateral flow assays (Cornelissen et al. 2016; Sheet et al. 2016), nucleotide sequencing/next-generation sequencing (NGS) (Oultram et al. 2017), and matrix-assisted laser desorption ionization time-of-flight mass spectrometry (MALDI-TOF MS) (Barreiro et al. 2017). Haptoglobin (acute phase protein) is a diagnostic biomarker widely used for evaluation of bovine mastitis (Kalmus et al. 2013). In a study, for early, sensitive and rapid detection of haptoglobin at clinically relevant amounts in milk, magnetite nanoparticles (MNPs)-based label-free chemiluminescence bioassay was demonstrated and resulted into quantitative detection of haptoglobin within a range of 1 pg/mL to 1 μg/mL with a detection limit of 0.89 pg/mL (Nirala et al. 2020).

Several studies have explored the oxidative status of the animals with inflammation of the mammary gland in cows (Kleczkowski et al. 2017). The inflammatory response associated with mastitis involves the release of several inflammatory mediators as well as reactive oxygen species (Turk et al. 2017). Mastitis has been found associated with high concentration of inflammatory and oxidative mediators. Significant changes have been observed in the levels of serum interleukins, tumour necrosis factor (TNF), acid glycoprotein (alpha-1 AG), and haptoglobin (Kleczkowski et al. 2017).

Cow serum proteome was evaluated previously to analyse differential protein expression in subclinical and clinical mastitis. The comparative study has helped to estimate the systemic inflammatory and oxidative stress response in case of subclinical and clinical mastitis (Turk et al. 2012). Findings from the study indicate that the inflammatory protein, vitronectin is over-expressed in both subclinical and clinical mastitis. Therefore, vitronectin is an important mediator in the onset of mastitis and can be considered as a valuable biomarker for diagnosis of the subclinical mastitis (Turk et al. 2012). Similarly, in another study, serum paraoxonase-1 activity was measured in clinical and subclinical mastitis in order to analyse systemic inflammatory and oxidative stress responses (Kovačić et al. 2019). The findings from the study indicate that paraoxonase-1 activity was significantly lower in animals with clinical and subclinical mastitis as compared to the control group (Kovačić et al. 2019). We can conclude that the subclinical mastitis induced oxidative stress and inflammatory reaction significantly lowered the paraoxonase-1 activity in blood and milk of affected cows (Nedić et al. 2019). Therefore, paraoxonase-1 activity could be considered as a potential biomarker for diagnosing subclinical mastitis.

Virulence of the causative agent determines the mammary glandular response to the pathogens that are invading the gland. In addition, the microbial environment as well as the host has great influence on the infectivity (relative) of the causative agent. Thus, epidemiology of mastitis changes in relation to the pathogens, host factors and environment (Klaas and Zadoks 2018). Although, the cause along with epidemiology of this particular inflammatory condition of the mammary gland is well established in food animals, but the challenge for diagnosing the condition with accuracy remains very much relevant (Sordillo 2011). For several pathogens causing mastitis in bovine, the routes of transmission along with sources as well as prognosis can be understood well by conducting studies based on molecular epidemiology. The mechanism of host adaptation can be understood in a better way by understanding the evolution of pathogens for evading host defenses (Zadoks et al. 2011). Moreover, by identifying the allelic profiles of the virulence or house-keeping genes, the persistence of pathogens can be assessed in a more effective way and in this regard molecular epidemiological studies play a great role. In this context, it is important to note that the development of multilocus sequence typing (MLST) scheme has further aided to these studies on molecular epidemiology (Sordillo 2011; Shibata et al. 2014). Recently, Internet of Things (IOT) has been used for detection of mastitis and foot and mouth disease (FMD) which could help in a better way in significant reduction of these diseses because of the use of Neural Networks and smart sensors (Vyas et al. 2019). This can diminish the very bad quality of milk produced in the cows; as a result, it can reduce the processing cost of dairies, thus benefiting the Agriculture and Dairy Industries in various economical ways.

## 6. Advances in treatment of mastitis

An efficient and effective mastitis control programme requires the early detection of infection by understanding the pathogenesis, discovering new sensitive tests for early screening, adopting good managemental practices to reduce the chance of transmission and preventing the uninfected ones. The control programme must include the strategic use of antimicrobials to curtail the problems of antibiotic residue in milk and antimicrobial resistance (Ruegg et al. 2017a). Prior to drug therapy, the main cause of udder infection needs to be clarified. The affections of the teat or udder, like teat fistula, leaky teat, teat spider, and udder wounds need immediate attention. Since such affections break the effective barrier and tend to expose the teat canal or udder to the external microbes; therefore, the earliest rectification is needed. In a study, cattle teat surface surgical affection number was significantly higher as compared to the other locations as well as the number of teat surgical affection in buffaloes (Nabil et al. 2018). Not all the affections of teat and udder required treatment. Out of total 282 cases of cattle and buffalo, 24% of the cases were left untreated, while 73.8% of the affected animals were treated by medical and surgical methods (Nabil et al. 2018). Most prevention activities focus on milking time as it is the prime period for new IMIs (Keefe 2012). The health and hygiene of dairy cows can be improved by practicing disinfection of the teat both before and after milking along with complete milk-out (Keefe 2012; Yu et al. 2017). The occurrence of clinical and subclinical mastitis is more frequently observed in heifers in early lactation than cows because of their managemental and physiological state. Improved prepartum managerial strategies in the arena of environmental and animal hygiene, *viz*., use of teat sealants and antiseptics, vector control, isolation of heifers from older cows and restricted feeding of mastitic milk to calves is recommended to control heifer mastitis (McDougall et al. 2009a). Prepartum treatment of heifers offers much higher cure rate, no milk loss and minimal risk of antibiotic residues; however lowered SCC and high milk production is not necessarily achieved in all herds (Borm et al. 2006). Moreover, reproductive performance of preparturient heifers was not affected significantly during the first 200 days of the first lactation after intramammary treatment (Borm et al. 2006). As per report, the lactational interventions in subclinical IMIs of dairy cows resulted in less transmission rates of *S. uberis*, *S. dysgalactiae* and *E. coli* with few flare-ups along with low IMI associated costs but this did not halt the transmission rate of *S. aureus*. Therefore, in dairy herds lactational treatment preceded by managemental practices are necessary to boost the udder health (van den Borne et al. 2010).

Successful treatment of clinical mastitis depends on several factors: antimicrobial treatment, causal agent identification, parity, stage of lactation, history of previous SCC, clinical mastitis and other systemic diseases (Steeneveld et al. 2011). Organic farmers in the USA treat the clinical mastitis cases using various alternative therapies, *viz*., homeopathy, botanicals, vitamin supplements and whey-based products, as restrictions imposed by the process of organic certification, like no usage of antimicrobials or hormones, usage of organic feeds, stress-free husbandry practices, which resulted in very few options left for control of mastitis in organic farms (Ruegg 2009). In a study under Dutch circumstances, cow specific treatment recommended for clinical mastitis was not found beneficial economically (Steeneveld et al. 2011). However, herd specific interventions like cow specific treatment and culling strategies against subclinical and clinical IMIs may prove highly cost effective in management of mastitis (Gussmann et al. 2019). The management of mastitis involves both preventive and therapeutic strategies, and is primarily based on antibiotic therapy. However, the recent approaches used for treating mastitis involves the use of natural therapeutics, such as zeolites and propolis could serve as an alternative to antibiotic therapy (Benić et al. 2018).

### 6.1. Antibiotic therapy

Antibiotics are frequently administered as a preventive measure to mastitis during the dry period. Dry cow therapy using antimicrobials is allowed as prophylactic measure among livestock species. Selection of antibiotics for the treatment of clinical mastitis should be based on history, etiology, antibiotic sensitivity profile and most importantly, it should be based on recommended therapeutic principles.

The pathogens isolated from the mastitis milk have been reported to exhibit wide spectrum of antibiotic susceptibility. In a study conducted in the Zenica region in Bosnia and Herzegovina, the highest antimicrobial resistance was observed against benzylpenicillin (56.3%) and oxytetracycline (46.2%) (Burović 2020). The *in vitro* antimicrobial susceptibility pattern of *T. pyogenes* infections was studied among domestic animals. The antibiotics such as florfenicol, cefoperazone, cephalexin, and ceftiofur were found to be effective while high resistance rates were observed against trimethoprim-sulfamethoxazole, norfloxacin, and tetracycline (Ribeiro et al. 2015).

With the emergence of antibiotic resistance, selection of antibiotics for management of mastitis should be based on the culture and sensitivity results rather than empirical therapy (Tiwari et al. 2013). Another important drawback of antibiotic therapy is its ability to produce antibiotic residues in milk that could be detrimental to the consumer’s health (Oliver and Murinda 2012; Gomes and Henriques 2016). These antibiotic residues are found to be stable for quite some time and can lead to adverse effects on consumers in addition to resistance (Kurjogi et al. 2019). As per report, the use of antibiotics, *viz*., oxytetracycline, amoxicillin and ciprofloxacin in cows resulted in presence of antibiotics residue both in raw and boiled milk at different time intervals and thus suggests the strict maintenance of withdrawal period of antibiotics in order to reduce its risk post treatment (Anika et al. 2019). Even though antibiotics are used extensively for the treatment of mastitis without even considering the severity of disease, most cases of non-severe forms of clinical mastitis will not be benefited from such uncontrolled usage. Such cases should be managed using alternative strategies (Ruegg 2017b). The current recommended strategies in managing clinical mastitis due to Gram-positive agents are directed against individual organisms by targeted antimicrobial therapy. Such therapeutic protocols provide sufficient time for the other cases to get spontaneously cured (Ruegg 2017b). Combination therapy of antibiotics that involve multiple routes of administration like systemic and intramammary route enhances the clinical cure rate. This may be due to the higher antimicrobial concentration in milk and mammary tissues (Lima et al. 2018b).

Selection of antibiotics based on culture and sensitivity does not guarantee 100% efficacy in clinical cases. This is because of the disparity in the results of *in vitro* antibiotic sensitivity and the failure of those sensitive antibiotics in the clinical cases. The mastitis caused by *S. aureus* is susceptible to a variety of antibiotics *in vitro* but due to the peculiar biology of staphylococci along with their adaptation to the bovine host environment, development of microabscesses and biofilm-formation, some of the antibiotic agents become ineffective clinically (Rainard et al. 2018). There is a need for critical and careful interpretation of laboratory results to avoid antibiotic therapy of staphylococci without taking into consideration the clinical relevance in suspected mastitis cases in bovine species to ensure the optimal and appropriate use of antibiotics (Wald et al. 2019). A wide variation in the antibiotic susceptibility spectrum has been observed throughout the world. This can further complicate the outcome of treatment with antibiotics. Timely novel compounds need to be evaluated for mastitis prevention and cure. Glycolic acid-based and iodine-based postmilking barrier teat disinfectants were evaluated by Lago et al. (2016) for prevention of new IMIs in dairy cattle. Glycolic acid-based disinfectants were found to be non-inferior to iodine-based disinfectant and were safe and effective as postmilking teat disinfectants. They reduced the incidence and new IMI (NIMI) by around 17%; however, SCC and teat conditioning were not affected. For the prevention of naturally occurring new intramammary infections and clinical mastitis, Martins et al. (2017) evaluated disinfectants with high free iodine and barrier quality in dairy cows. They noted that those teat disinfectants having barrier properties and higher free iodine content when used at postmilking time were able to reduce the risk of clinical mastitis; however, effect on new infections were observed at only weekly intervals. Those animals disinfected with barrier postmilking teat disinfectant (BAR) had 46% lower odds for clinical mastitis than those disinfected with nonbarrier postmilking teat disinfectant (NBAR). Such odds for NIMI were 54 and 37% lower with NBAR disinfected group at 8 and 16 weeks, respectively, compared to BAR disinfected group.

Several studies involving *in vitro* antibiotic sensitivity of microorganisms isolated from bovine mastitis reported varying degrees of antibiotic resistance of isolates globally (León-Galván et al. 2015; Su et al. 2016; Shah et al. 2019). The bovine mastitis isolates from Mexico showed a resistance pattern to antibiotics mainly penicillin, clindamycin, and cefotaxime (León-Galván et al. 2015). In a study conducted in southern Taiwan, all of the *E. coli* isolates from the milk of cows with clinical mastitis showed resistance to cloxacillin whereas some of the isolates showed resistance to tetracycline, neomycin, gentamycin, ampicillin, ceftriaxone, cefotaxime, and ceftazidime (Su et al. 2016). Methicillin resistance genes were common in the isolates of *S. aureus* obtained from the bovine mastitis cases in India and Thailand (Shah et al. 2019). There are even reports of simultaneous occurrence of multiple resistant microbial isolates obtained from clinical cases of bovine mastitis in India. Fourth generation cephalosporin was found to be slightly better than the conventional cloxacillin and ampicillin combination to treat subclinical *S. agalactiae* mastitis (Rossi et al. 2019). Oxytetracycline could be used as first line of treatment in acute *E. coli* mastitis in cattle; however, anticipation of its effectiveness is not possible (Shinozuka et al. 2019). In case of mild to moderate *E. coli* mastitis, antibiotics should be avoided whereas in severe cases antibiotics like fluoroquinolones and cephalosporins through parenteral route is recommended to prevent bacteremia-associated risk (Suojala et al. 2013). All the variations in the susceptibility spectrum along with the emergence of resistance affect the efficacy of antibiotics in mastitis treatment. Ceftizoxime has been found effective in treatment of acute staphylococcal mastitis in Indian cross-bred cows (Buragohain et al. 2019). Such variations in the susceptibility spectrum will in turn affect the response of microorganism towards the antimicrobial agents. Although extensive use of antibiotics for prevention of mastitis may contribute to resistance and antibiotic residues in milk but the advantages of the antibiotics prevail over the above said disadvantages (Oliver and Murinda 2012).

Steele and McDougall (2014) evaluated effect of prolonged therapy of subclinical mastitis by penethamate hydriodide (PH) over bacteriological cure proportion and SCC in dairy cows. Therapy by PH resulted in increase in bacteriological cure proportion, decreased percentage of glands infected after therapy, and decreased SCC. Thus, the PH helps in curing intrammary infections in subclinical mastitis; however, effect is lower in older cows and those infected with *S. aureus*, especially when resistant to penicillin. Fuenzalida and Ruegg (2019) found that intramammary ceftiofur had no improvement effect on culling rate, milk yield or production, or SCC when used for the therapy of non-severe culture-negative clinical mastitis cases. Intramammalial (IMM) ceftiofur aggravated *K. pneumoniae* mastitis and resulted in chronic intramammary infection and worst clinical outcomes. In case of *E. coli* mastitis IMM ceftiofur is not deemed to be necessary (Fuenzalida and Ruegg 2019).

However, currently, no effective option other than antibiotics is available. Intramammary infusion of tilmicosin at drying may be an effective option to prevent NIMI mainly caused by environmental streptococci and coagulase-negative staphylococci (Dingwell et al. 2002). An experimental study showed that intramammary infusion of ceftiofur prevents *S. uberis* infection. Such an effect was more pronounced in an extended therepy of 8 days compared to the 2-day treatment option (Oliver et al. 2004). A comparative study of the two antibiotics including tylosin base (5 g injected 3 times at 24-h intervals; n = 306) and penethamate hydriodide (5 g injected 3 times at 24-h intervals; n = 289) showed no significant differences in cure rates of mastitis; 79.8% *vs.* 82.0% of cows treated, respectively (McDougall et al. 2007). However, there are many factors that determine the success rate of the antibiotics in mastitis including the microbe types, udder environment, and milking type (machine/hand). In addition to the parenteral antibiotic, the addition of non-steroidal anti-inflammatory drugs resulted in lower SCC, reduced milk yield losses, improved clinical outcomes, and reduced culling rates as compared with antibiotic therapy alone (McDougall et al. 2009b). In *E. coli* mastitis, non-steroidal anti-inflammatory drugs (NSAIDs) were reported to be effective and recommended for supportive therapy in management of clinical mastitis (Suojala et al. 2013).

Among the different etiological agents that are responsible for clinical mastitis, *S. aureus* is the one among few that can cause a big headache for the clinician/veterinarian due to its peculiar pathogenesis, contagiousness, environmental persistence, skin or mucosal colonization, and the poor response to current therapeutic agents (Rainard et al. 2018). Eradication of *S. agalactiae* can rapidly be achieved through treatment program but the same is often unsuccessful in case of *S. aureus* (Kefee 2012). It is reported that higher occurrence of biofilm formation among the methicillin-resistant strains of *S. aureus* can augment its pathogenicity (Shah et al. 2019). All these factors make *S. aureus* the most difficult agent to be eradiccated from the herds. Effectiveness of antibiotic therapy has been long gone in *S. aureus* induced mastitis, probably due to its over usage (Park et al. 2012) or due to the induction and persistence of biofilm-associated antibiotic resistance in mastitis due to *S. aureus* (Babra et al. 2013). That might be the reason for the continuous attempt to develop vaccines against mastitis caused by *S. aureus*, which is yet to become successful (Côté-Gravel and Malouin 2019). A recent report suggested that NZ2114 derivative peptide, H18R (H2) can be used as a safe and potential candidate for treating *S. aureus* induced mastitis (Wang et al. 2019). A study suggested that nasal immunization against *S. aureus* associated mastitis in bovines results in an increase level of anti-*S. aureus* specific IgA antibodies in milk and a negative correlation between anti-*S. aureus* specific IgA antibodies and numbers of *S. aureus* counts in the treated udder which may lead to the use of nasal vaccines in *S. aureus* associated mastitis (Nagasawa et al. 2019)

For the successful implementation of antibiotic usage in reduction of mastitis, there should be an increase in the diagnostic effort so that unnecessary antibiotic usage can be prevented (Krömker and Leimbach 2017). Prudent use of antibiotics with proper awareness, scientific basis of reduction in antibiotic use, and also a legal requirement for judicious use should be exercised keeping in view the cost of treatment of mastitis and potential benefits (Doehring and Sundrum 2019). Antimicrobial therapy of cows with recently acquired subclinical mastitis (RASCM) should be done only in exceptional cases due to current focus on reduced and judicious use of antimicrobials in animal husbandry therapeutic practices (van den Borne et al. 2019). There is a need for continuous monitoring of antibiotic resistance of major bacterial pathogens causing mastitis in cows, and also an urgent need for harmonization of methodology and interpretations (Chehabi et al. 2019). Intramammary preparation of ceftiofur hydrochloride showed no significant difference on overall clinical cure, bacteriological cure, and new infection when compared to the preparation containing tetracycline, neomycin, bacitracin, and prednisolone when used for treating non-severe clinical mastitis in dairy cows (Cortinhas et al. 2016).

Skoulikas et al. (2018) used pirlimycin for an extended therapy against intrammary infections in heifers caused by *S. aureus*. Significantly higher cure rate (64.8%) was noted in treated group compared to control group (34.1%). Thus, they suggest that higher cure rates for *S. aureus* intramammary infections in heifers can be achieved when the extended treatment protocol is applied immediately after calving. Extending the period of intramammary therapy may help in preventing clinical failures but may not have an effect on cure proportion, somatic cell count, or new infection rate. In one such study, McDougall et al. (2019) have reported that intramammary treatment with a combination of amoxicillin, clavulanic acid, and prednisolone over short (3 times at 12-h intervals) and long periods (5 times at 12-h intervals) significantly reduced rate of clinical failures but had no effect on cure proportion, SCC, or new infection rate. Mastitis caused by *Prototheca* spp. are very difficult to manage as there are no effective therapies (Alves et al. 2017; Dos Anjos et al. 2019). The *in vitro* algicidal effect of guanidine was previously studied on *Prototheca zopfii* genotype 2 strains isolated clinical and subclinical bovine mastitis. Guanidine exhibited algicidal action at low concentrations can be considered an alternative disinfectant, antiseptic for cleaning, chemical dry therapy of bovine teats or even as an intramammary therapeutic agent (Alves et al. 2017).

### 6.2. Bacteriophage therapy

Treatment of biofilm forming bacteria poses a great challenge due to their resistance against conventional antibiotics. In such instances, other modes of therapy have to be selected for successful elimination of the etiological agent. Bacteriophages are a group of viruses that have the ability to infect and kill bacteria (Haq et al. 2012; Tiwari et al. 2014). They have the inherent ability to target and destroy specific bacterium and also the capacity to replicate exponentially which makes them a potential candidate against pathogenic bacteria (Carson et al. 2010). Several bacteriophages have been isolated and studied for their potential in eliminating pathogenic bacteria associated with mastitis (Dias et al. 2013; Porter et al. 2016; Amiri Fahliyani et al. 2018; Varela-Ortiz et al. 2018). Several potential candidates have been identified for bacteriophage therapy against *S. aureus* (Dias et al. 2013; Varela-Ortiz et al. 2018)*, Klebsiella oxytoca* (Amiri Fahliyani et al. 2018) and *E. coli* (Porter et al. 2016). Since all of the evaluations were based on *in vitro* studies, further *in vivo* studies have to be conducted to prove their efficacy in clinical cases. Even though bacteriophages can be considered as effective against microorganisms, most of them lack environmental stability and have to be stored and handled under certain special conditions, which limits their usage (Skurnik et al. 2007). Dias et al. (2013) reported that the isolated bacteriophages had features like thermostability and high lytic potential, which made them suitable candidates against the *S. aureus* antibiotic resistant strains. All of the thermostable phages maintained high titer even after incubation at a temperature of 100 °C for 5 minutes (Dias et al. 2013). Another modification in the bacteriophage therapy is the utilization of a phage cocktail instead of a single bacteriophage. When phage cocktail was used for the treatment of *S. aureus*-induced mastitis in mouse model, it was found superior to either of the individual phages used alone (Geng et al. 2019). The mice treated with phage cocktail were able to maintain the highest intramammary phage titer compared to other groups and its efficacy was comparable to that produced by the antibiotic ceftiofur sodium. Phages induce phage-specific humoral response and memory, which can hamper therapeutic success (Krut and Bekeredjian-Ding 2018). The lytic efficacy of bacteriophage mixture containing of three phages, STA1.ST29, EB1.ST11, and EB1.ST27 was evaluated against *S. aureus* isolates. The significant reduction in the *S. aureus* germ density indicated the therapeutic potential of bacteriophage therapy and requires further validation by performing *in vivo* studies (Titze et al. 2020). Further studies are required to prove the *in vivo* efficacy of bacteriophage therapy in managing bovine mastitis.

### 6.3. Bacteriophage endolysins

Another potential therapeutic agent, called endolysins derived from the bacteriophages are effective against Gram-positive pathogens. They are the proteins that allow the phage to escape from the bacterial cell during the phage lytic cycle by degrading the peptidoglycan layer of bacterial cell wall (Breyne et al. 2017). Fenton et al. (2013) reported a novel bacteriophage-derived peptidase, CHAP_K_ to be an effective biocidal agent that can be used for the rapid disruption of biofilm-forming staphylococci. The *in vitro* efficacy of CHAP_K_ suggests that it can be included in the teat-dip solution to prevent the colonization of *S. aureus* over the surface of udder skin (Fenton et al. 2013). Several other peptidoglycan hydrolases are also found to control and treat infections caused by staphylococcal group of bacteria. Some of these anti-staphylococcal peptidoglycan hydrolases include lysostaphin, LasA, ALE-1, broth lysate, CsCl, LytM, AtlA, AtlE, LysK, SAL-1, MV-L, ClyS, and LysH5 (Gill et al. 2006; Szweda et al. 2012).

### 6.4. Antimicrobial peptides

Antimicrobial peptides (AMPs) are new generation antibiotics that destroy invading microorganisms and have a major role in the innate immune mechanism (Pieterse and Todorov 2010; Moravej et al. 2018). They have broad-spectrum activity against several Gram-positive and Gram-negative bacteria including some of the drug-resistant strains. AMPs produce synergism when used along with conventional antibiotics (Chung and Khanum 2017). The AMPs of multicellular organisms such as defensins and cathelicidins have a major role in the innate immunity of vertebrates, whereas the AMPs of unicellular organisms like bacteriocins enable them to suppress competitor species (Sang and Blecha 2008). It also encourages the cells to stimulate angiogenesis, produce chemokines, accelerate wound healing process, and influence apoptosis in multicellular organisms (Moravej et al. 2018). The mechanism by which AMPs produce immunity against microorganism is a primitive one (Chung and Khanum 2017). The therapeutic application of AMPs is very much limited in the present scenario due to the short half-life, high production cost, enzymatic degradation, and cytotoxic effects on the eukaryotic cells (Moravej et al. 2018).

The β-defensins are AMPs that come under the “defensin” family. They are considered as the best known genetically encoded antimicrobials and act as the first line of defense against IMIs occurring in dairy cattle (Gurao et al. 2017). In an *in vitro* study, bovine umbilical vein endothelial cell lines were analyzed for expression of different antimicrobial peptides when stimulated with *S. aureus* or LPS. The result suggests that the bovine endothelial cells play a major role during bacterial infection through β-defensin production (Alva-Murillo et al. 2012). Cathelicidins are AMPs produced by neutrophils. The infiltrating neutrophils present in mastitis milk release massive amounts of cathelicidin by degranulation resulting in high concentration of AMPs in mastitic milk (Cubeddu et al. 2017). They have the ability to directly control infection and regulate host defenses, which enables them to have broad-spectrum antimicrobial activity directed against bacteria, fungi, and enveloped viruses (Langer et al. 2017; Young-Speirs et al. 2018). The AMP cathelicidin are present in the mammary epithelial cells of animals affected with IMI but not in animals with healthy udder tissues. This makes it a sensitive and specific mastitis marker (Cubeddu et al. 2017). The bovine cathelicidins BMAP-27 and BMAP-28 were evaluated against the field isolates of *S. aureus* and *E. coli* obtained from diseased dairy cows suffering from clinical and subclinical mastitis. The result suggests that bovine cathelicidins are potential candidates to be included in the treatment strategies against bacterial mastitis in dairy cattle (Langer et al. 2017). Further evaluation and characterization of the antimicrobial properties of bovine peptides are necessary to identify susceptible bacterial species.

Bacteriocins are antimicrobial peptides synthesized by the ribosome and are secreted by various Gram-positive and Gram-negative bacteria (Desriac et al. 2010). They are considered as an alternative to antibiotics, which are currently used for the prevention and treatment of mastitis (Godoy-Santos et al. 2019). Bacteriocins can also be considered as the future of treating infections like mastitis by a targeted, effective and safe approach (Ahmad et al. 2017). This is one of the solutions for preventing the emergence of antibiotic resistant strains of microorganisms. Nisin and Bovicin HC5 are the two major examples of bacteriocins that are reported to have well established therapeutic effect against microorganisms responsible for mastitis (Castelani et al. 2019; Godoy-Santos et al. 2019). Nisin is bacteriocin produced by *Lactococcus lactis* ssp. *lactis*. They have antimicrobial activity against several Gram-positive and foodborne bacteria (Cao et al. 2007; Wu et al. 2007). In a study conducted to evaluate the efficacy of Nisin against drug-resistant *Staphylococcus* spp. isolated from bovine mastitis, it was found to have *in vitro* bactericidal activity (Castelani et al. 2019). Nisin at certain dose (2,500,000 IU × 3 days; Intramammary) shows variability in antimicrobial activity in subclinical mastitis; bacteriological cure rate being more for *S. agalactiae* (90.1%), followed by coagulase-negative staphylococci (58.8%) and *S. aureus* (50%) (Wu et al. 2007). In a comparative study against gentamicin, a comparable cure rate of clinical mastitis cases with Nisin was reported (Cao et al. 2007). Bovicin HC5 is a bacteriocin produced by the ruminal bacterium *Streptococcus equinus* HC5 (Mantovani et al. 2001). A study was conducted to evaluate the efficacy of the ruminal bacteriocin, bovicin HC5 against the most prevalent microorganisms found in contagious udder infections. Bovicin HC5 was effective in inhibiting most of the streptococcal and staphylococcal strains but was ineffective against *E. coli* strains; thus, making it less effective alternative of antibiotics (Godoy-Santos et al. 2019). The resistance against AMP is a serious threat that limits its potential use as an antimicrobial agent. Several diverse mechanisms are responsible for the development of resistance against AMPs. Some of them involve production of proteases by the bacteria, modification of cell surface charge, altering the membrane fluidity, and activation of efflux pumps (Moravej et al. 2018).

### 6.5. Probiotics

Probiotics are getting more popularity in the treatment of several inflammatory conditions and diseases. Several microorganisms have been evaluated for their probiotic activity but lactic acid bacteria are the major group of probiotic organisms (Dhama et al. 2017). Lactic acid bacteria can provide protection against mastitis when they are used as feed supplements, teat dip, and intramammary inoculation due to their potent immunomodulatory activity (Pellegrino et al. 2017; Yu et al. 2017; Rainard and Foucras 2018). The lactic acid bacteria colonize the udder and prevent mastitis by forming a protective biofilm, which inhibits the growth of mastitis-causing pathogens (Rainard and Foucras 2018; Wallis et al. 2018). The gut microbiome and their metabolites play an important role in maintaining the health of dairy cow. Lipopolysaccharide (LPS) and short-chain fatty acids are the two major products of gut microbes. Increased production of rumen-derived LPS results in its entry into the blood circulation; when it reaches the mammary gland, it increases blood-barrier permeability resulting in inflammation of mammary gland, whereas short-chain fatty acids, the fermentation product of rumen microbiota, have shown a protective effect on the mammary gland (Hu et al. 2019). The incorporation of lactic acid bacteria in the animal feed can be considered as an effective tool for the prevention of bovine mastitis (Pellegrino et al. 2017).

Several strains of lactic acid bacteria exhibited biofilm formation during *in vitro* evaluation. The extent of biofilm formation strongly depended upon the strain of bacteria, surface charge and medium (Wallis et al. 2018). Three strains of lactic acid bacteria, *Lactobacillus brevis* 1595, *L. brevis* 1597 and *L. plantarum* 1610 among the total of 165 isolates obtained from sampling of the teat canal exhibited high colonization capacities of bovine mammary epithelial cells. These identified strains have the potential to compete against mastitis pathogens in colonizing mammary gland (Bouchard et al. 2015). The two strains of lactic acid bacteria isolated from bovine milk *L. lactis* subsp. *lactis* CRL 1655 and *L. perolens* CRL 1724 exhibited inhibitory activity against bovine mastitis pathogens by means of co-aggregation and by adhering to the bovine teat canal epithelial cells during *in vitro* evaluation (Pellegrino et al. 2019). Adhesion of the lactic acid bacteria to the epithelial surface will help in preventing the invasion of pathogenic bacteria. There exists a negative correlation between the amount of *Lactobacillus* bacteria and the mastitis pathogens in the mastitic milk. This may indicate an active protective effect exhibited by the *Lactobacillus* bacteria against mastitis (Qiao et al. 2015). There is significant relationship between the health of bovine udder and *Lactobacillus* count of the milk. The lactic acid bacteria *L. casei* BL23 was found to have the ability to modulate innate immune response of bovine mammary epithelial cells that are infected with *S. aureus* bacteria. It reduced the expression of several pro-inflammatory cytokines such as IL-6, IL-8, IL-1α, IL-1β and tumour necrosis factor-alpha (TNF-α) in *S. aureus*-stimulated bovine mammary epithelial cells indicating potent anti-inflammatory activity (Souza et al. 2018). Similarly, the *L. casei* strain has the ability to inhibit the invasion of bovine mammary epithelial cells by *S. aureus* bovine strains that caused clinical mastitis by inhibiting the adhesion and/or internalization of *S. aureus* (Bouchard et al. 2013).

Intramammary inoculation of *L. lactis* subsp. *lactis* CRL 1655 and *L. perolens* CRL 1724 during dry-off period increased the amount of immunoglobulin (IgG isotypes) in blood and milk. The results suggest that lactic acid bacteria have potent immunomodulatory activity produced by stimulating local and systemic immune response (Pellegrino et al. 2017). Intramammary infusion of lactic acid bacteria exhibited pro-inflammatory activity that induced an influx of neutrophils into the milk both during lactation and at drying-off (Rainard and Foucras 2018). Teat dip using a novel probiotic lactobacilli-based teat disinfectant was found to be superior to the commercial disinfectant in reducing somatic cell count (Yu et al. 2017). The reduction in the mastitis-associated bacteria is due to the improved microbial environment of the teat. Such environment friendly lactic acid bacteria disinfectants can replace commercially available chemical disinfectants. There is no strong scientific foundation that supports the use probiotics to treat mastitis in dairy cattle but they have the ability to alter the teat apex microbiota, which helps to prevent the colonization of teat canal by mastitis causing pathogens (Rainard and Foucras 2018). Probiotics along with or without repeated milk-out are not recommended for efficient management of clinical mastitis in dairy lactating cows (Francoz et al. 2017).

Apart from probiotics, microbial extracts can also be utilized to treat mastits. One study showed actinomycetes-derived crude extracts (Caat1-54 and CaatP5-8) contained antimicrobial metabolites that inhibited bacterial growth obtained from clinical and subclinical mastitis. It was demonstrated that these extracts presented an *in vitro* antimicrobial activity against isolates of *S. aureus*, *S. chromogenes*, *S. dysgalactiae*, and *S. uberis* (Leite et al. 2018).

### 6.6. Herbal therapy

Herbal therapy is a promising area in treatment of mastitis as no adverse effect is associated with it. Ethno-veterinary medicine is a branch of veterinary medicine that deals with the treatment of diseases with herbal preparations (Tiwari et al. 2018). Medicinal plants can be used as an alternative therapeutic option or as an adjunct agent in managing bovine mastitis. They can be used as an anti-bacterial, anti-inflammatory, and immunomodulatory agent for the treatment of mastitis (Mushtaq et al. 2018). The anti-inflammatory and anti-bacterial effects of Chinese herbs have been utilized effectively in the treatment of bovine mastitis (Muluye et al. 2014; Yang et al. 2019). They can also be used as a replacement to antibiotic and anti-pyretic agents that are generally used in the treatment of mastitis (Muluye et al. 2014). Ranjith et al. (2018) reported that the methanolic extracts of herbal preparation containing *Diploclisia glaucescens* leaf and rhizomes of *Curcuma longa* in equal proportions produced analgesic activity along with anti-inflammatory activity. The analgesic activity of the herbal extract was found to be comparable to that of ibuprofen and indomethacin (Ranjith et al. 2018). Herbal therapy includes different routes of administration based on the type of formulation. Among them, topical route (Hase et al. 2013), oral administration (Dash et al. 2016) and intramammary routes (Yang et al. 2019) are the commonly used methods. In a comparative study conducted to evaluate the efficacy of homeopathic complex therapy, herbal therapy (Neem seed extract) and antibiotic therapy for the treatment of subclinical mastitis in dairy buffaloes, it was found that treatment with antibiotics were having superior efficacy over the herbal (Neem seed extract) and homeopathic complex therapy groups. When cost factor was also taken into consideration, the herbal therapy was found to be the cheapest (Younus et al. 2018). Hence, it can be effectively used as an adjunct to antibiotics in the treatment of clinical mastitis without causing much alteration in the cost factor. Some herbal extracts may have anti-inflammatory and antioxidant values that help in curing inflammation of udder and minimizing oxidative stress. *Moringa* extract has been shown to ameliorate inflammatory mediators and increase antioxidant systems in bovine udder epithelial cells. It inhibited proinflammatory cytokine (TNF-α, IL-1β, and IL-6) expression, cyclooxygenase-2 expression and downregulated NF-κβ, upregulated heme-oxygenase-1, NAD(P)H; and quinone oxidoreductase-1 besides the extract of *Moringa* plant increased expression of casein proteins (Cheng et al. 2019).

There are several plant species used for prevention and control of bovine mastitis in southern Brazil due to their anti-inflammatory, immuno-modulatory and antibiotic effects (Avancini et al. 2008; Xu et al. 2015). Leaves, bark, bulb and aerial parts were used for the preparation of herbal medicine. Plant species like *Achillea millefolium, Allium sativum, Alternanthera brasiliana, Baccharis trimera, Chenopodium ambrosioides, Cuphea carthagenensis, Foeniculum vulgare, Phytolacca dioica, Sambucus nigra, Sida rhombifolia, Solanum mauritianum, Atractylodis macrocephalae* Koidz and *Solidago chilensis,* were used orally, among which *Alternanthera brasiliana, Baccharis trimera* and *Sambucus nigra* were also used as topical agents. *Ocimum basilicum* and *Parapiptadenia rigida* were the two plant species that were used by intramammary route in bovine mastitis (Avancini et al. 2008). *Staphylococcus epidermidis* is one of the main causes of medical device-related infections and bovine mastitis owing to its biofilm-forming abilities. *Oxytropis glabra* is a Fabaceae species that is widely used as a Chinese herbal formulation in Western China. The *in vitro* studies conducted to evaluate the effect of *O. glabra* decoction on the *S. epidermidis* biofilm formation identified potential inhibiting mechanism that can be further explored in developing newer drugs against biofilm-associated infections (Ren et al. 2020).

In a study to evaluate the efficacy of *Ocimum sanctum* leaf juice as supportive therapy for the management of induced chronic staphylococcal mastitis, it was found that leaf extract had significant bio-enhancing and anti-oxidant activities, which can be effectively utilized by combining with antibiotics (Dash et al. 2016). So rather than going for herbal therapy as a single agent in managing clinical mastitis, superior results can be obtained if they are included in the treatment protocol as an adjunct along with other modes of therapy. In a recent study conducted to evaluate the *in vitro* anti-bacterial activity of ethyl acetate extract of the plant *Terminalia chebula* against the molecularly identified isolates of *S. aureus, E. coli, Pseudomonas aeruginosa,* and *Bacillus megaterium*; it was found that 500 µg/mL concentration of the extract had the same antibacterial efficacy as that of standard amoxicillin (Kher et al. 2019). This finding gives an insight into the potential of herbal extracts in replacing antibiotics as the single agent in managing clinical mastitis.

The botanical preparations like PHYTO-MAST® contains ingredients (Thymol, methyl salicycate, glycyrrhizin, and α-pinene) considered within the “Generally Recognized as Safe” United States Food and Drug Administration (FDA). The ingredients have anti-inflammatory, analgesic, antipyretic and antiseptic properties and can be effective in treating mastitis (McPhee et al. 2011). However, one of the studies failed to show any therapeutic effect upon 3 days intramammary application repeated at 12 h-interval (Pinedo et al. 2013).

**Figure 1.**
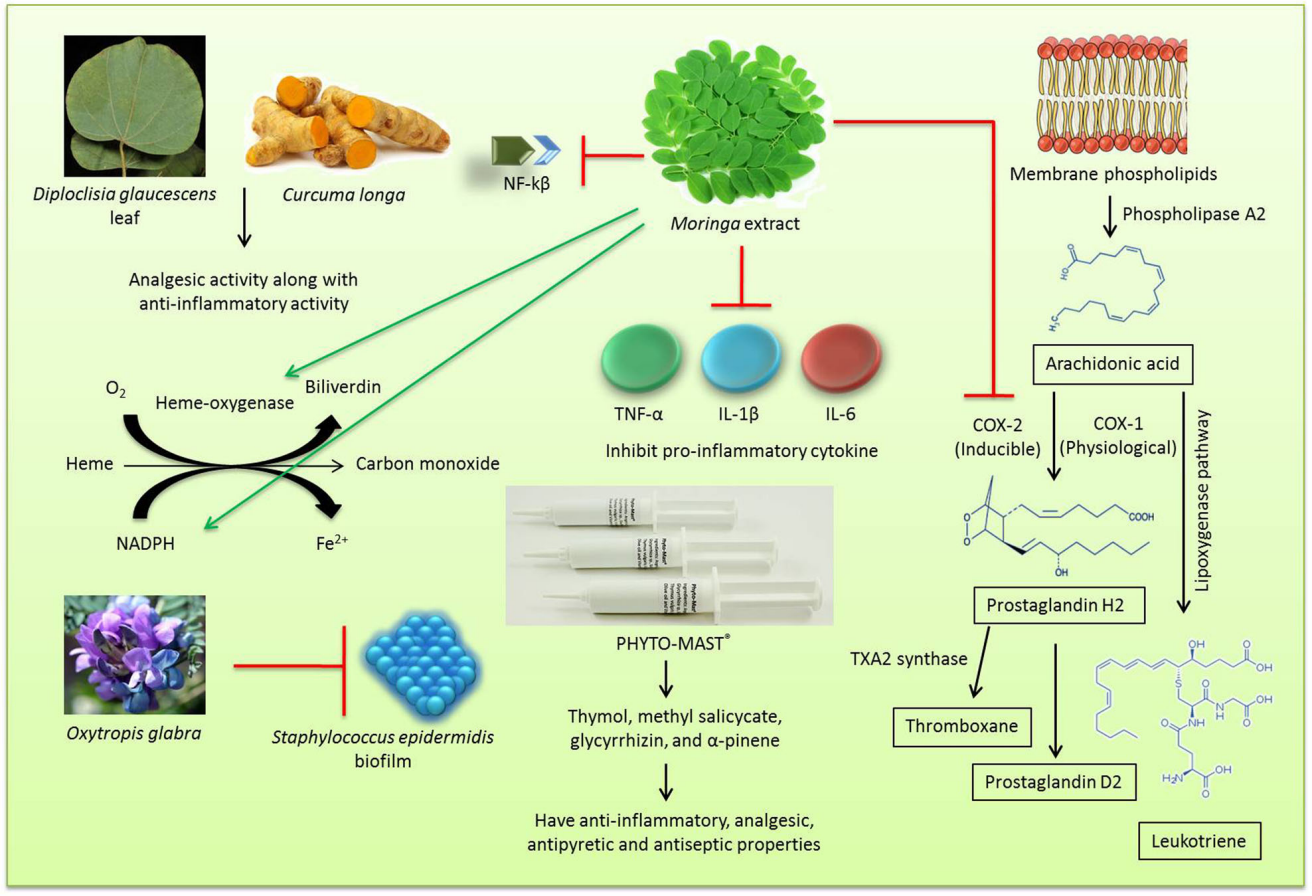
Herbal therapy for treatment of mastitis. Different medicinal herbs possess anti-inflammatory and analgesic properties.

An overview on the role and mode of action of herbal therapy for the treatment of mastitis is presented in Figure 1.

### 6.7. Immunotherapy

Immunotherapy is an alternative, immunologically-based treatment for mastitis. Leitner et al. (2013) treated 32 mastitic cows naturally infected with *E. coli*, *S. dysgalactiae* or coagulase-negative staphylococci (CNS) using microbeads carrying specific antibodies to the mastitis causing bacteria and an enhancer of phagocytosis, termed Y-complex in comparison to treatments with sulfadiazine + trimethoprim or procaine penicillin + streptomycin (BA) (8 cows), or a non-steroidal anti-inflammatory drug (NSAID) (19 cows). The Y-complex was as effective as antibiotics, and superior to NSAID, in eliminating the bacteria. However, the use of NSAID like meloxicam in addition to the antibiotics improved the fertility of the cows (McDougall et al. 2016). The Y-complex caused less discarded milk as compared to antibiotic treatment group. *E. coli* and *S. aureus* have proven to be involved in modulating the immune response in bovine mastitis. As per evidence, challenge with *E. coli* induces a vigorous immune response resulting into systematic immunomodulation in *in vivo* models, whereas *S. aureus* induces a weak response (Petzl et al. 2018). A single dose of interleukin-2 (IL-2) injected into the skin region drained by the supramammary lymph node 3–5 days after calving induced significant increase of several milk markers related to white blood cell and epithelial cell functions including SCC, serum amyloid A (SAA), lactoferrin and NAGase (Zecconi et al. 2009). The increased concentration of milk markers suggested also an activity of IL-2 on epithelial cells, resulting in a higher resistance to invading pathogens. Addition of IL-2 to a regular dry cow antibiotic targeting *S. aureus* enhanced the effectiveness of the treatment. However, abortion was observed in 7.5% of the cows that received the IL-2 (Texasdairymatters.org). Immunostimulants are also an active area for mastitis management. Infusion of extract of *Saccharomyces cerevisae* yeast into mammary gland during the dry period caused an increase in the activity of immune cells in the gland, which could respond immediately to a new infection. It was suggested that this treatment could minimize the risk of a new IMI during the periparturient period (Texasdairymatters.org). The therapeutic potential of egg yolk immunoglobulins (IgYs) for treating mastitis was evident from several *in vitro* studies (Zhen et al. 2008; Wang et al. 2011). Specific IgY can be produced against *E. coli* and *S. aureus* by immunizing hens with formaldehyde killed bacteria in a long-standing immunization response. The immunoglobulin isolated from yolks enhanced phagocytic activity against mastitis-causing bacteria, indicating its potential use as therapeutic agent in mastitis treatment (Zhen et al. 2008). In order to study the protective effect of antibodies on *S. uberis* mastitis, Almeida et al. (2015) evaluated anti-recombinant *S. uberis* adhesion molecule (SUAM) antibodies in dairy cows against *S. uberis* intramammary infections. These cows were infused with *S. uberis* UT888 opsonized with affinity purified anti-rSUAM antibodies or hyperimmune sera as test animals against non-opsonized *S. uberis* UT888 as control animals. Clinical symptoms of mastitis were mild to undetectable in test dairy cows. Further test animals showed lower milk bacterial counts, and less infected mammary quarters compared to control animals. This indicates better protection against infection of *S. uberis* by these antibodies through prevention of adherence and inhibition of entry into mammary glands thereby helping in clearance of pathogens especially *S. uberis*. Colonization of microbes is reduced; hence less IMIs.

**Figure 2.**
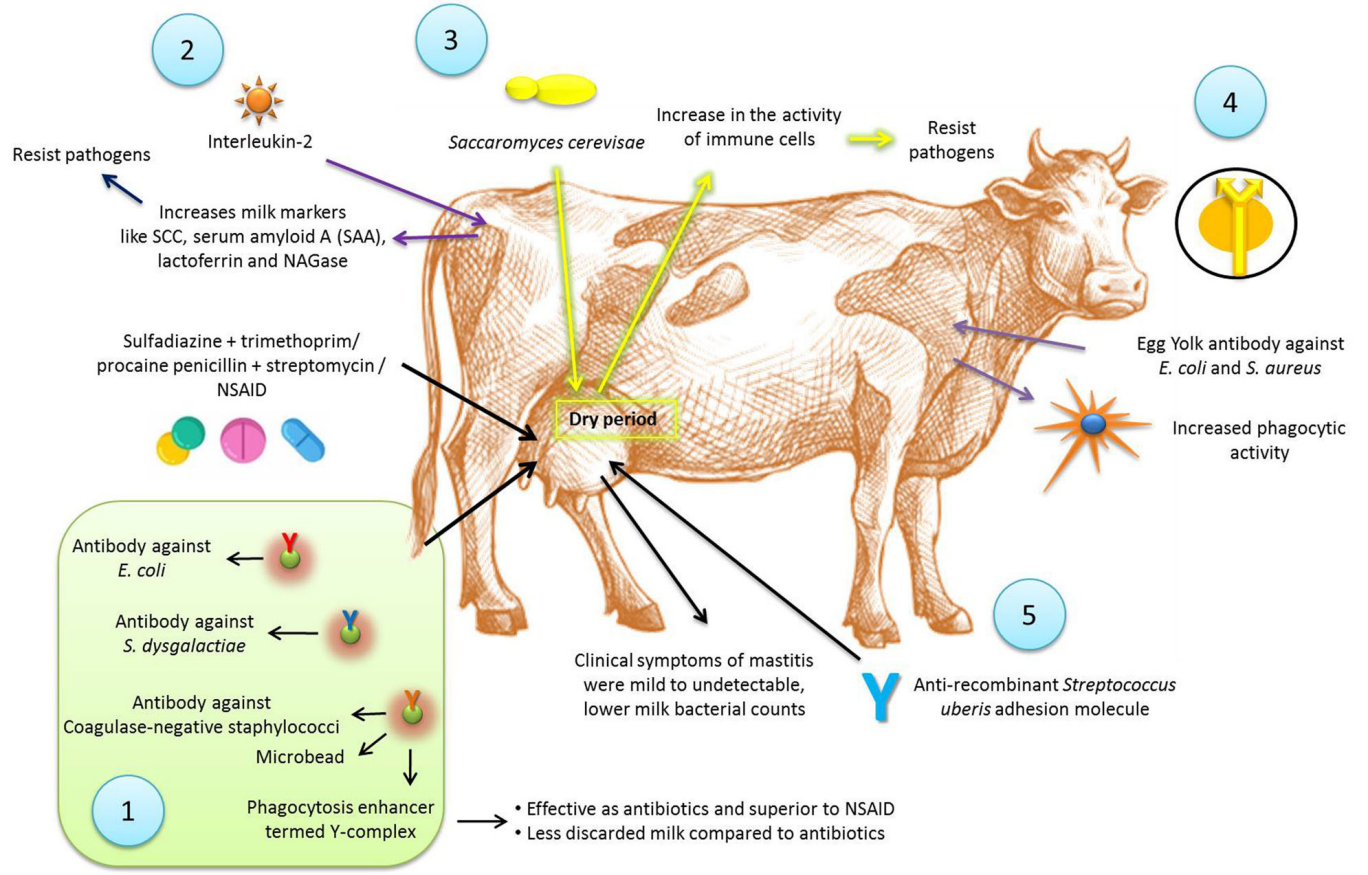
Use of immunotherapy for treatment of mastitis. 1. Microbeads carrying specific antibodies to the mastitis causing bacteria and an enhancer of phagocytosis, termed Y-complex was showing effects similar to sulfadiazine + trimethoprim or procaine penicillin + streptomycin or NSAIDs. 2. Interleukin-2 injection was showing increase of several milk markers related to white blood cell and epithelial cell functions including SCC, serum amyloid A (SAA), lactoferrin and NAGase. 3. Infusion of extract of *Saccharomyces cerevisae* yeast into mammary gland during the dry period caused an increase in the activity of immune cells in the gland, which could respond immediately to a new infection. 4. Specific IgY can be produced against *Escherichia coli* and *Staphylococcus aureus* increased phagocytic activity. 5. Use of anti-recombinant *Streptococcus uberis* adhesion molecule showed reduced clinical symptoms of mastitis, lower milk bacterial counts and lower infected quarters.

An overview on the application and mechanisms of action of immunotherapy for the treatment of mastitis is presented in Figure 2.

### 6.8. Nanoparticle-based therapy

Nanoparticle technology is another area that is currently developing as a delivery technique for antimicrobial agents and other drugs (Gomes and Henriques 2016). Different types of nanoparticles have already been evaluated for the treatment of mastitis with positive results (Castelani et al. 2019; Kalińska et al. 2019; Orellano et al. 2019; Pinheiro Machado et al. 2019). Nanoparticle formulations will enhance the uptake of active compound by phagocytes and thereby improving its antibacterial activity (Gruet et al. 2001). They are found to be effective against several multi-drug resistant bacteria that pose a great threat to the society (Yu et al. 2018; Castelani et al. 2019). Bovine mastitis caused by *S. aureus* is very difficult to manage using conventional therapies due to its efficient pathogenesis, characteristic facultative intracellular parasitism, biofilm formation, and emerging antimicrobial resistance. Hence, nanoparticle-based therapeutic techniques like liposomes, nanogels, polymeric nanoparticles, inorganic nanoparticles, and solid lipid nanoparticles are gaining popularity as excellent tools for managing *S. aureus* mastitis (Algharib et al. 2020). Honey, when used along with gold nanoparticles produced significant *in vitro* anti-microbial activities against methicillin-resistant (MRSA) and vancomycin-resistant (VRSA) coagulase-positive *S. aureus* mastitis strains (Omara 2017). Nanoparticles when used at high doses will cause impairment of several organs resulting in pathological changes (Elbehiry et al. 2019). Further studies are required to identify the biological effects of nanoparticles. Some of the nanoparticles, like silver and copper nanoparticles (Kalińska et al. 2019), chitosan nanoparticles (Orellano et al. 2019), propolis nanoparticles (Pinheiro Machado et al. 2019) and cationic nisin-lipid nanoparticles (Castelani et al. 2019) have been reported to show positive results in managing mastitis. The antibiotics that act by inhibiting protein synthesis exhibited significant synergistic effect when used along with silver nanoparticles (Kazemi et al. 2014). The anti-bacterial activity of antibiotics like tilmicosin and amoxicillin can be enhanced by nanoformulation (Yang et al. 2009; Zhu et al. 2018). The solid lipid nanoparticles of tilmicosin exhibited prolonged and enhanced anti-bacterial activity against *S. aureus* and *S. agalactiae* (Zhu et al. 2018). Amoxicillin nanoparticles enhanced the post-antibiotic effect and reduced the dosing interval when used against pathogenic bacteria causing bovine mastitis (Yang et al. 2009). The *S. aureus* strains isolated from mastitis cases were found to be susceptible to silver and gold nanoparticles. Gold nanoparticles were superior to silver nanoparticles since *S. aureus* strains develop resistance less frequently against gold nanoparticles (Elbehiry et al. 2019). The therapeutic efficacy of α-linolenic acid-based intramammary nanosuspension (ALA-NS) was evaluated for the treatment of subclinical mastitis. Treatment with ALA-NS was found to be associated with significantly decreased expression of SREBP-1c (sterol response element-binding protein-1c), NF-κB-p65 (nuclear factor kappa-light-chain-enhancer of activated B cells), and UCHL-1 (ubiquitin carboxyl-terminal hydrolase-1) along with the decrease of total microbial count and somatic cell count (Yadav et al. 2020).

Chitosan nanoparticles (Ch-NPs) are reported to have great therapeutic potential for bovine mastitis (Orellano et al. 2019). Commercially available metal nanoparticles like silver and copper nanoparticles decreased the *in vitro* viability of *S. aureus* and *E. coli* without producing toxic effect on mammary gland (Kalińska et al. 2019). As per report, sonochemically synthesized capped zinc oxide nanoparticles showed higher antibacterial activity than particles synthesized by auto-combustion method in the intramammary treatment of clinical bovine mastitis caused by *S. aureus*, *E. coli*, and *K. pneumonia*; thereby suggesting its potential regarding mastitis control (Hozyen et al. 2019). The anti-bacterial action of nanoparticles has to be further validated by *in vivo* studies. The ability of mastitis causing opportunistic pathogen *E. coli* to form biofilm is responsible for the development of antibiotic resistance (Yu et al. 2018). Plant derived nanoparticles are getting popularity for managing mastitis (Chaitanya Kumar et al. 2013; Yu et al. 2018). The silver-nanoparticle-decorated quercetin nanoparticles produced by combining silver nanoparticles with plant-derived quercetin exhibited strong anti-bacterial and anti-biofilm activity against multi-drug resistant *E. coli* strains isolated from dairy cattle with mastitis (Yu et al. 2018). Curcumin is the polyphenol obtained from turmeric and has low oral bioavailability due to its rapid elimination from the body, which limits its anti-inflammatory activity. The nanoformulation of curcumin can improve its oral bioavailability and can act by reducing the pro-inflammatory mediators in *S. aureus*-infected mammary tissue mouse model (Suresh et al. 2018). Similarly, the silver nanoparticles derived from aloin, a major constituent of *Aloe vera* exhibited potent anti-bacterial activity against *S. aureus* in experimental murine mastitis model (Chaitanya Kumar et al. 2013). Further research is required to evaluate the application of nanoparticles in mastitis therapy. Effect of combination of chitosan and cloxacillin on planktonic cultures, bacterial biofilms and intracellular growth in udder cells have been studied (Breser et al. 2018). It was shown that combined approach of using chitosan and cloxacillin inhibited the formation of biofilm, improved clearance of already formed biofilm. Besides, it decreased viability of intracellular bacteria, which is believed to be due to enhanced release of IL-6 by affected mammary epithelial cells. Thus, this therapy can be a novel method of prevention of mastitis in a safe, effective and contamination-free mode especially overcoming problems related to multi-drug resistant bacteria.

### 6.9. Stem cell therapy

The stem cells of bovine mammary epithelial cells play a major role in maintaining the udder health. Such stem/progenitor cells can be utilized for treating mastitis induced structural/cytological defects in the bovine udder (Sharma and Jeong 2013). Mesenchymal stem cells have anti-bacterial activity due to the ability to produce certain factors that inhibit bacterial growth (Yuan et al. 2014; Cahuascanco et al. 2019). The bone marrow derived mesenchymal stromal cells also have *in vivo* anti-bacterial activity against methicillin-resistant *S. aureus* in rat model. The enhanced bacterial clearance is produced either by enhancing the innate immune response or by promoting the expression of certain anti-microbial peptides (Yuan et al. 2014). The conditioned medium produced from mesenchymal stem cells derived from bovine fetal bone marrow and adipose tissue exhibited *in vitro* antibacterial activity against *S. Aureus*, which is mediated by β-defensin 4 A and NK-lysine 1 activity (Cahuascanco et al. 2019). Mesenchymal stem cells also have the ability to modulate inflammatory responses, but the precise mechanism behind this activity is yet to be identified (Yuan et al. 2014). This property can be potentially utilized in managing bovine mastitis. Human mesenchymal stem cells exhibit broad-spectrum antimicrobial activity which is mediated by expression of the enzyme indoleamine 2,3-dioxygenase (IDO). On the contrary, murine mesenchymal stem cells failed to express IDO and hence lacked anti-bacterial activity (Meisel et al. 2011). Such species-specific difference in the anti-bacterial activity of stem cells has to be considered before utilizing it in mastitis therapy.

Allogeneic adipose tissue mesenchymal stem cells (AT-MSCs)-based intramammary therapy was evaluated in dairy cows experimentally infected with *S. aureus.* Intramammary inoculation of allogeneic AT-MSCs (2.5 × 10*^7^*) reduced the bacterial count of milk in clinical mastitis compared to the untreated cows. It is also important to note that the intramammary inoculation was not associated with clinical or immunological response in healthy cows (Peralta et al. 2020). Bovine mammary stem cell therapy can be used for regenerating mammary tissues by means of either repairing or replacing the damaged tissue. The stem cells have the ability to differentiate into epithelial, myoepithelial and/or cuboidal/columnar cells of the udder tissue. By utilizing bovine mammary stem cells, we can reduce the risk of rejection and the possible side effects (Sharma and Jeong 2013). Since the mammary stem cells are responsible for the growth, renewal and turnover of mammary epithelial cells, they can be utilized for tissue repair and improving milk yield (Capuco et al. 2012). Further researches on isolation and characterization of mammary stem cells are necessary for attaining a greater understanding of the normal epithelial cell development in mammary tissue (Sharma and Jeong 2013). Advancement in this area is mandatory before proceeding to treat mastitis with bovine mammary stem cell therapy.

Even though there are several well-established treatment techniques along with a great number of emerging techniques, treatment of mastitis will always be a challenge to the clinician/veterinarian due to its broad spectrum of etiological factors along with the wide variety of clinical manifestations. Farmer’s role and perception towards management of mastitis are very important, in this context an online questionnaire regarding mastitis completed by 290 full time Swedish dairy farmers emphasized the role, knowledge and skills of farmers in mastitis management (Lind et al. 2019).

### 6.10. Native secretory factors

Lactoferrin (Lf), a natural whey protein of mammary gland origin, carry many biological functions. Among various functions, limited anti-bacterial and anti-inflammatory activities are prominent that can aid in treating mastitis. Anti-bacterial effects of Lf arise through iron binding required for bacterial growth, besides, being cationic in nature. Its weak anti-bacterial activities can be potentiated in combination with several antibiotics like penicillin G (Petitclerc et al. 2007). A study demonstrated that Lf increases the inhibitory activity of penicillin up to 4-fold in most penicillin-susceptible *S. aureus* strains. The potentiated inhibitory activity of Lf increased from 4- to 16-fold in penicillin-resistant strains. The inhibitory effect of Lf arises by blocking the beta-lactamase activity in *S. aureus* strains as its gene transcription is dramatically repressed in the presence of Lf (Lacasse et al. 2008). Apart from utilization along with the antibiotics, the bovine lactoferricin gene (*LFcinB*) has been cloned into PiggyBac Transposon vector. Such a vector clone has been transfected into the bovine mammary epithelial cells resulting in three-fold increase in LFcinB secretion. The secreted LFcinB had strong antibacterial activity against *S. aureus* and *E. coli* with 14.0 ± 1.0 mm and 18.0 ± 1.5 mm zone of inhibition, respectively (Sharma et al. 2017).Phospholipases A2 under *in vitro* studies have been demonstrated to have anti-inflammatory property. Such an activity, however, is seen after cells are treated with the LPS or live bacteria (*E. coli* or *S. aureus*). There arises significant improvement in cell viability after phospholipases A2 application with reduction in IL-8; suggesting its role in prevention of cell membrane damage. In compliance to the results, the *in vivo* application of a single PLA2G1B in chronic cases infected with *S. dysgalactiae* has been undertaken. The treatment was reported to completely clear inflammation and bacteria, demonstrating its potential to cure subclinical mastitis. However, coagulase-negative staphylococci infection was unaffected and may have been due to the formation of a resistant biofilm (Seroussi et al. 2018).

Homeopathy is an effective option in some cases of mastitis but not under all circumstances. As such similar to the antibiotics, homeopathy may show effectiveness against the particular microbes. Various studies have compared the effect of the antimicrobial agent against homeopathy (with a placebo control). It was shown that all these treatment options had limited effectiveness (Werner et al. 2010). In culture-positive cases, suboptimal bacteriological cure (60-81%) was seen with antibiotic treatment that had better effective cure rates than individualized homeopathic therapy (33-43%). These cure rates were comparable to those of placebo (45-47%). The cytological cure level in all three treatment methods were similarly ineffective being 2-21% in antibiotic treated; 0-8% in individualised homeopathy and 3-13% in placebo (Keller and Sundrum 2018). Even some of the studies have shown a significantly lower cure rate in clinical mastitis with the homeopathy compared to the antibiotic treatment (Williamson and Lacy-Hulbert 2014; Ebert et al. 2017). Homeopathic, non-anti-microbial and other alternative conventional treatments are not advised in case of clinical mastitis of dairy lactating cows (Francoz et al. 2017).

## 7. Management of mastitis

A better package of practice for dairy cattle can prevent problems like mastitis in the farm. Instead of treating the IMIs condition, it is feasible to prevent the occurrence of mastitis. Hence, good management practices are the integral part of successful prevention of mastitis. Several management practices that include from the selection of the cattle to nutritional aspects are dealt with in the following section.

### 7.1. Genetic selection

Resistance to mastitis can be improved by means of genetic selection as it has been found that there is significant genetic variation between individual cows. Hence, the resistance can be improved by means of sire selection, which results in long-term improvement of herd health (Weigel and Shook 2018). In a study conducted among Italian Jersey cows, it was found that the SCCs of milk were higher in cows having deep udders, loose udder attachments, weak ligaments, and long teats. So, if traits like milk production, fat yield, protein yield, and fore-udder attachment, udder support and udder depth are included in the selection index, the udder health can be increased by genetic improvement (Bobbo et al. 2019). Transcription factors: PP53, SP1, ligands INS, IFNG, EGF and protein kinases, like MAPK1, MAPK14, AKT1 were identified as important upstream regulators, whereas protein kinases MAPK3, MAPK8, MAPK14, ligands VEGF-A, IL-10, an extracellular protein MMP-2 and mitochondrial membrane protein BCL-2 were identified as key downstream targets of differentially expressed genes, which have key functions in immune responses, inflammation or mastitis, which may form strategies to improve the treatment of mastitis in cows caused by bacteria, *e.g., E. coli* (Sharifi et al. 2019) and others. The screening of more ancient breeds that have been least manipulated for milk yield, like *Bos indicus* for coding region of β-defensin genes can be used as a tool for selecting more resistant animals (Gurao et al. 2017). There is a recent effort at an international level to improve the phenotypic and genotypic data sets that can improve the selection for mastitis resistance. The accuracy of utilizing breeding values in selecting animals for mastitis resistance can be enhanced by utilizing cutting-edge technologies (Martin et al. 2018).

### 7.2. Nutrition

There exists a greater relationship between the nutrition of animal and resistance of mammary tissue to infection. This is credited to the ability of nutrients to supply antioxidant function that improves the immune resistance against infections (Erskine 1993). Trace minerals like selenium, copper, zinc, and vitamins like vitamin A/β-carotene, and vitamin E can affect the udder health (O'Rourke 2009). Injectable trace mineral supplement containing zinc, manganese, selenium, and copper reduced the incidence of chronic clinical mastitis in dairy cows with elevated SCC (Ceballos-Marquez et al. 2010; Ganda et al. 2016). Those dairy cattle that have negative energy balance are predisposed to ketosis, and those animals having clinical ketosis are having two-fold increase in the risk of clinical mastitis (O'Rourke 2009). Copper supplementation in dairy cattle challenged with intramammary *E. coli* lowered the bacterial count and SCC. There was a significant decrease in the severity of an *E. coli* infection but duration of infection remained unchanged (Scaletti et al. 2003). Selenium produces anti-inflammatory activity by downregulating inflammatory mediators (Ma et al. 2018; Wang et al. 2018). The dietary supplementation of selenium in mice produced anti-inflammatory activity by inhibiting the activation of the NALP3 inflammasome and nuclear factor-kappaB/mitogen activated protein kinase (NF-κB/MAPK) pathway in *S. aureus* induced mastitis. There was a decrease in the expression of IL-1β, TNF-α, NRLP3, caspase-1, and ASC in the mouse mammary tissue corresponding to the increasing selenium concentrations (Ma et al. 2018). The anti–inflammatory activity is also reported due to increased miR-146a expression. When miR-146a inhibitors are used, the anti-inflammatory effects of selenium was found to be suppressed (Sun et al. 2017). Selenium also down-regulated the inflammatory mediators TNF-α, IL-1β and IL-6 gene expressions which is mediated by the Toll-like receptor 2 (TLR2), NF-κB and MAPK signaling pathways in bovine mammary epithelial cells stimulated by *S. aureus* (Wang et al. 2018). In a cattle study, selenium supplementation in precalving cows did not result in reduction of the incidence of new intramammary infection and clinical mastitis during the balance of the first month of lactation. However, pasture-based heifers that were injected with barium selenate before calving, and fed diets of Se/day in precalving and/or during lactation, no cases of clinical mastitis were observed in the first month of lactation (Ceballos-Marquez et al. 2010). The combined supplementation of selenium and vitamin E, however, has been shown to improve resistance to the mastitis through elevated neutrophil α-tocopherol concentrations during the periparturient period. The elevated levels of neutrophil and α-tocopherol may negate the suppressed intracellular killing of bacteria by neutrophils that are commonly observed at calving (Hogan et al. 1993; Smith et al. 1997).

Supplementation of vitamins A, D3, E, and H can help in the recovery from subclinical mastitis by increasing the expressison of host defense genes. Vitamin D activates innate immune responses of bovine monocytes and alters the oxidants-anti-oxidants balance to normal (Merriman et al. 2017, 2018). The vitamins increased the total anti-oxidant capacity and glutathione peroxidase activity along with reduction in total oxidant capacity, nitric oxide and catalase levels (Dimri et al. 2013). After intramammary treatment with vitamin D, decreased bacterial counts has been documented as the vitamin is known for its anti-microbial activity along with decreased inflammatory response (Nelson et al. 2018). Vitamin E and selenium supplementation enhances the phagocytic activity, thereby decreasing clinical mastitis risk (Heinrichs et al. 2009). Addition of vitamin E and selenium as an adjunct to antibiotic therapy can improve the cellular defense along with reduction of SCC in mastitic animals, when compared to that of antibiotic alone (Mukherjee 2008). Adequate quantity of vitamin E is obtained by the intake of fresh grass and grass silage. Sustained-release rumen bolus formulation containing vitamin E and selenium can be used for supplementation in case of deficiency (Hemingway 1999). Dietary supplementation of anti-oxidants such as vitamins A and E can have beneficial effect on the health of mammary gland in dairy cattle (Erskine 1993). Retinoic acid, a derivative of vitamin A attenuates inflammatory response in LPS-induced rat mastitis model by suppressing the TLR-4/NF- κB signalling pathway (Gu et al. 2010). Dietary supplements like fatty acids and cholecalciferol regulate adhesion gene expression as well as bacteria internalization in non-professional phagocytic cells. This could lead to development of anti-virulence agents for control of *S. aureus* mastitis in bovine (Frutis-Murillo et al. 2019).

Dietary supplementation of zeolite clinoptilolite has been found to alter the chemical composition of milk and udder health in dairy cows (Ðuričić et al. 2017). In another study, the impact of dietary vibroactivated and micronized clinoptilolite feeding in the incidence of intramammary infections was evaluated in dairy cows. It was observed that the antibacterial, antioxidative, immunostimulating and detoxifying potential of vibroactivated and micronized zeolite clinoptilolite reduced the incidence of intramammary infections (Đuričić et al. 2020). Strategies have to be developed for using targeted nutrition to obtain positive host response in managing diseases. Such a technique can limit the use of antimicrobial drugs that has side effects like drug residues and possibility of developing drug-resistance (Sordillo 2016). Further studies are required to define the relationship between plane of nutrition and mastitis resistance.

### 7.3. Dry cow therapy and lactation therapy

Dry cow therapy and lactation therapy are the two protocols of antibiotic therapy used in managing mastitis. Lactation therapy involves the treatment of mastitis with antibiotics during the period of lactation (Tiwari et al. 2013). This system of treatment lacks practical utility due to the high cost of therapy along with poor efficacy (Pyörälä 2009). In a study conducted to evaluate the long-term efficacy of antibiotic therapy of recently acquired subclinical mastitis during the lactation period, it was found that there is no beneficial long-term effects (van den Borne et al. 2019). It was found that for the treatment of subclinical mastitis due to contagious pathogens, antimicrobial treatment during lactation period can be profitable due to the prevention of intramammary infection in susceptible cows (van den Borne et al. 2010). Hence, as an exception, the lactation therapy can be used for the treatment of subclinical mastitis caused by contagious pathogens (van den Borne et al. 2010; 2019).

Dry cow therapy involves the treatment of dairy cattle during the dry period. Dry cow therapy aimed to extinguish existing IMIs and control the new infections during the dry period (Berry and Hillerton 2002a; Derakhshani et al. 2018). This period is critical in controlling the mastitis at the herd level. This is because IMIs when treated during dry period result in higher cure rates (Halasa et al. 2009; Cameron et al. 2015). This can be attributed to the absence of dilution effect produced by the milk during lactation period that reduces the effectiveness of antibiotic therapy against the etiological agent. Another important factor that makes dry cow therapy critical is the fact that the rate of occurrence of NIMI is high in dry period when compared to other stages of lactation (Halasa et al. 2009). In a study, effect of dry cow therapy was compared with no treatment, in which no clinical mastitis cases were reported during dry period in treated group whereas a significant number of clinical mastitis cases along with new IMIs after calving were reported in untreated group (Berry and Hillerton 2002a). Selective dry cow therapy is a variant of dry cow therapy that involves antimicrobial selection based on the culture and sensitivity results. This reduces the unnecessary use of antimicrobials in dairy production (Cameron et al. 2015). Cameron et al. (2015) evaluated petrifilm-based on-farm culture system selective antimicrobial treatment with respect to blanket dry cow therapy. They did not find any significant difference between the two regimes with respect to quarter-level cure risk, NIMI risk over the dry period, the risk of IMI at calving, and the risk of clinical mastitis in the first 120 day suggesting that selection of dry cow therapy on petrifilm-based on-farm culture system is as efficient as blanket dry cow therapy in preventing IMI during dry period and clinical mastitis after calving. In a study, udder quarters of clinically healthy Holstein dairy cows were subjected to dry cow therapy using penicillin G and novobiocin along with teat sealant followed by evaluation of dynamics of the microbiota of mammary secretions and teat canal. It was found that this approach has limited success in extinguishing the significant proportion of microbes during the dry period (Derakhshani et al. 2018). The SCC obtained from the milk sample can be considered as a convenient and accurate method in choosing the animals for selective dry cow therapy (Zecconi et al. 2019). Based on the study conducted by Zecconi et al. (2019) the recommended threshold for the selection of cows for dry cow therapy is based on the SCC values of 100,000 cells/mL for primiparous cows and of 200,000 cells/mL for pluriparous cows. Use of ciprofloxacin along with internal teat sealant as a dry off protocol was found effective as it revealed 24 and 31% lower risk of overall NIMIs and NIMIs due to most prevalent pathogens in comparison to traditional dry therapy **(**de Magalhães Rodrigues Martins et al. 2019). For minimizing antibiotic usage, Vasquez et al. (2018) evaluated an algorithm against the antibiotic based dry cow therapy. This algorithm is culture independent and helps in deciding the selected antibiotics for dry cow therapy. By using this algorithm, about 60% of the dry-cow antibiotic was reduced and there was no effect on health and production; however new infection risk quarters experiencing new infections, milk production, linear scores, culling events, or clinical mastitis events did not differ significantly between algorithm and antibiotic used groups.

Mohammadsadegh (2018) compared an intramammary infusion of tilmicosin with respect to cloxacillin, which is used as a traditional intramammary infusion for dry cow therapy. Total bacteriological cure rates (45.0%) and new infection rates (43.3%) were lower in tilmicosin treated cows compared to cloxacillin treated cows (78.0%, 56.6%); however SCC was higher (6.732 × 10^5^ ± 3.124 × 10^5^ *vs.* 5.025 × 10^5^ ± 2.935 × 10^5^). Tilmicosin showed potent activity against IMI by *S. aureus*, less against *Corynebacterium bovis* and no effect against *S. agalactiae* (Mohammadsadegh 2018).

### 7.4. Teat sealants

The opening of teat canal before calving is an important predisposing factor responsible for the occurrence of heifer mastitis. Most of the teat canals under study were already open several months before calving (Kromker and Friedrich 2009). The risk of developing subclinical and clinical mastitis in heifers can be reduced by using internal teat canal sealant during pre-calving period (Parker et al. 2007). Internal teat sealant (ITS) when used in combination with antibiotic dry-cow therapy significantly reduced the SCC along with improved prevention of subclinical mastitis (Golder et al. 2016). In a study conducted to evaluate the efficacy of ITS in preventing NIMIs, it was found that 83% quarters retained the ITS plug till first milking after calving (Kabera et al. 2018). Several modifications are made in the composition of ITS for enhancing its ability to prevent mastitis (Compton et al. 2014; Serna-Cock and Pabon-Rodriguez 2016). The teat bio-sealant developed using *Weissella cibaria*, a lactic acid bacterium having probiotic activity exhibited anti-microbial activity against *S. aureus* and *S. agalactiae* (Serna-Cock and Pabon-Rodriguez 2016). A novel ITS containing bismuth subnitrate and chlorhexidine was found to reduce the incidence of NIMIs in adult cows and nulliparous heifers (Compton et al. 2014). In a study, effect of bismuth subnitrate teat canal sealant on existing IMIs was investigated in 1067 heifers (seasonally calving, pasture-fed dairy herds) and reduction in the prevalence of postcalving IMI by 65%, risk of NIMIs by 74% and the risk of new infection with *S. uberis* by 70% was reported indicating that ITS may be considered as a useful tool for successful management of mastitis in heifers during high risk peripartum period (Parker et al. 2008). In Ireland, use of a teat sealant along with intramammary cloxacillin resulted in reduced SCC and prevention of clinical mastitis during dry period (Berry and Hillerton 2002b).

The intramammary infections caused by environmental pathogens during early lactation can be prevented by using internal teat sealant along with long-acting dry cow antibiotics (Mutze et al. 2012). Treatment of cows with internal teat sealant is not likely to affect the transfer of colostral antibodies from cows to calves (Laven 2012). To minimize chances of predisposition to subclinical and clinical mastitis, combination of internal teat sealant and cephalonium dry cow therapy has been found more effective compared to cephalonium alone (Bates et al. 2016). It also indicates that mastitis can be prevented after calving by dry cow therapy plus teat sealents during dry period and upto calving. The administration of ITS can be done earlier than the current recommendation of 4 weeks before the planned start of calving without affecting its efficacy (Chambers and Newton 2019). The application of an external teat sealant during the pre-calving period reduces the prevalence of intramammary infections in pasture-fed dairy heifers (McDougall et al. 2008). The duration and variation of sealant adherence while using external teat sealants are dependent upon cow-level, quarter-level and herd management factors (Lim et al. 2007). Teat sealant alone may be beneficial in cows with better mastitis control programme as it helps in reducing SCC (McParland et al. 2019). For the prevention of clinical and subclinical mastitis in seasonal calving dairy cows, Runciman et al. (2010) evaluated 600 mg cloxacillin (CL) and 2.6 g bismuth subnitrate as internal teat sealant (ITS) after final milking of the season. Upto 21 day of lactation, rate of detection of clinical mastitis differed between various groups; however, after 21 day there was not much difference in this rate. Further CL + ITS helped in decreasing number of cases with clinical and subclinical mastitis, and also helped in decreasing this rate in cows with previous history of subclinical mastitis.

### 7.5. Acoustic pulse therapy

Acoustic pulse therapy (APT) also known as shockwave therapy utilizes the hand-held instrument that produces pulsing pressure waves. Such waves penetrate through the deeper tissues and can break scar tissue of the chronic wounds leading to revascularization. In a study that utilized APT in subclinical mastitis, a statistically significant population of the mastitis cattle (70.5%) could return to normal milk production as compared to control group (18.4%). The percentage of cows with log SCC of milk below 5.6 cells/mL was significantly higher in APT treated cows as compared to control. Even the isolated bacterial type infected cows were significantly cured by APT, though differences in the cure rate was reported with the type of bacteria involved. In the same study of clinical mastitis, APT cured 76.9% of the affected cows (n = 13) while only 18.7% were cured by the gold standard antibiotic therapy (Leitner et al. 2018). This therapy though appears promising but further extensive including the involved infectious agent types studies are desired to standardize the therapy. In addition, class IV laser and interferential therapy may be combined with APT to evaluate the healing potential.

## 8. Conclusion and future perspectives

Mastitis affects animal welfare and causes economic and production losses by deteriorating milk quality, reducing production performance, increased culling rate, cost of treatment, and due to mortality associated with per acute form of the disease. Several groups of microbial organisms can cause both clinical and subclinical forms of the disease. Subclinical mastitis is economically more important compared to clinical mastitis due to its ability to deteriorate milk quality to such a level that it cannot be detected grossly but will affect the overall quality. Several well established and economic conventional diagnostic techniques are available for the diagnosis of mastitis but lacks sensitivity and specificity. They cannot be used extensively in the current dairy production sector since they cannot provide rapid results. Recently developed advanced diagnostic tools used for diagnosis of etiological agents in mastitis are easy to use, rapid and sensitive but still lack considerable specificity. Such technique lacks economic sustainability due to the requirement of technical assistance, advanced equipment, and infrastructure facilities.

Once mastitis is diagnosed the main challenge for the veterinarian or the producer is to treat the animals in such a way that it will not deteriorate and become an economic burden to the production system. Several therapeutic strategies like antibiotics, vaccines, bacteriocins, herbal therapy, immunotherapy, and nanoparticle technology have been evaluated for efficacy in treating mastitis, but no single technique was found to be effective in controlling or treating the disease due to the variable response of etiological agents to the therapeutic techniques. Until now, antibiotics have been widely used as the sole therapeutic agent in managing mastitis, but with the emergence of bacterial resistance, which occurred due to the unsupervised use of antibiotics, several other treatment options are being explored. Development of an universal therapeutic agent/technique that can be considered as a replacement of the antibiotic therapy is the need of this century. Such a therapeutic agent/technique can solve the emerging problem of bacterial resistance. Further research should be directed towards the advanced therapeutic strategies, like bacteriocins and nanoparticle technology, which can offer a solution to the present situation. Diagnostic techniques and treatment modalities should be developed hand-in-hand so that early farm level diagnosis can be made accurately, which then can be combined along with specific therapy against the diagnosed microorganism that will enable the control and treatment of the mastitis effective.
